# Case Report: First case of percutaneous transhepatic cholangioscopy guided triple bridge drainage between multiple bile ducts for malignant hilar biliary obstruction

**DOI:** 10.3389/fonc.2025.1620937

**Published:** 2025-10-02

**Authors:** Gang Tang, Jie Zhang, Rui Chen, Jingyi Zhang, Rongxing Zhou

**Affiliations:** ^1^ Division of Biliary Tract Surgery, Department of General Surgery, West China Hospital, Sichuan University, Chengdu, Sichuan, China; ^2^ Department of Medical Ultrasound, West China Hospital, Sichuan University, Chengdu, China

**Keywords:** malignant hilar biliary obstruction, percutaneous transhepaticcholangioscopy, bridging technique, biliary drainage, case report

## Abstract

**Background:**

Biliary drainage for advanced malignant hilar biliary obstruction (MHBO) remains a significant challenge in current clinical practice.

**Case description:**

A 58-year-old male diagnosed with unresectable advanced intrahepatic cholangiocarcinoma with hilar obstruction and required palliative biliary drainage. Imaging revealed obstruction of the common bile duct, left hepatic duct, right anterior hepatic duct, and right posterior hepatic duct (Bismuth–Corlette type IV). Due to the failure of ERCP, we decided to bridge biliary drainage with percutaneous transhepatic cholangioscopy (PTCS) after multidisciplinary discussion. First, one-step PTCS was used to establish a channel between the skin and the right anterior hepatic duct. Then a puncture needle was used to puncture the right anterior hepatic duct to the distal common bile duct, and the first stent was inserted for bridging. Next, a puncture needle was used from the right anterior hepatic duct to the left hepatic duct and a second stent was inserted for bridging. Finally, a puncture needle was used to puncture the right anterior hepatic duct to the right posterior hepatic duct, and a third stent was inserted for bridging. Intraoperative X-ray examination with contrast agent injected through the sinus confirmed successful bridging. The jaundice disappeared a few days after surgery, and no post-procedure-related adverse events occurred.

**Conclusion:**

This case demonstrates that ultrasound-guided PTCS triple-bridge biliary drainage connecting multiple bile ducts is a feasible palliative option for MHBO and warrants further clinical investigation.

## 1 Introduction

Malignant hilar biliary obstruction (MHBO) can arise from intrahepatic and extrahepatic bile duct cancer, ampulla cancer, hepatocellular carcinoma, pancreatic cancer, or metastatic tumors from other primary sites (1, 2). MHBO often leads to obstructive jaundice and impaired liver function, and the prognosis of patients with unresectable MHBO is poor. In clinical practice, biliary drainage is widely used to relieve jaundice and improve quality of life (3, 4). Adequate biliary drainage is associated with prolonged survival (3, 5). However, effective biliary drainage in advanced MHBO (particularly Bismuth-Corlette type III–IV) remains a significant technical challenge (6, 7).

Percutaneous transhepatobiliary drainage (PTBD) and/or endoscopic retrograde cholangiopancreatography (ERCP) are well-known palliative biliary drainage strategies recommended by the guidelines of the European Society for Gastrointestinal Endoscopy (8). Unfortunately, the strategies currently available are difficult to achieve the desired drainage effect. Complicated MHBO often requires multiple PTBD. However, multiple PTBDs may increase the risk of complications and discomfort compared to unilateral PTBD. Biliary bridge drainage offers a potential alternative for complex MHBO. However, ultrasound guided hepaticogastrostomy bridge drainage can only establish a single bridge between two bile ducts (the left and right hepatic duct), and there is still a lack of technology to achieve multiple bridge drainage between multiple intrahepatic ducts for the treatment of multiple intrahepatic bile duct occlusion (6).

Herein, we report a novel technique using PTCS to achieve triple-bridge drainage among multiple hepatic ducts, enabling bilateral drainage in patients with Bismuth Corlette III and IV MHBO.

## 2 Case presentation

### 2.1 Patient presentation

A 58-year-old male presented with jaundice, no systemic disease, comorbidities, or prior abdominal surgery. There were no clinical manifestations related to cholangitis such as fever and abdominal pain. Laboratory tests revealed markedly elevated serum bilirubin levels: total bilirubin 537.7μmol/L and direct bilirubin 462.8μmol/L. CT showed advanced intrahepatic cholangiocarcinoma with hilar bile duct invasion, rendering the tumor unresectable. Obstruction was noted in the common bile duct, left hepatic duct, right anterior hepatic duct, and right posterior hepatic duct, corresponding to Bismuth-Corlette type IV (**Supplementary Figure S1**). The pathological diagnosis was intrahepatic cholangiocarcinoma.

### 2.2 Diagnostic findings and surgical treatment

The patient was diagnosed with advanced intrahepatic cholangiocarcinoma with hilar obstruction and required palliative biliary drainage. We decided to perform PTCS guided bridge drainage between multiple biliary ducts after multidisciplinary discussion.

One-step PTCS was adopted (9, 10) (**Supplementary Video 1**). Step 1: After successful general anesthesia through tracheal intubation, the patients were tilted to the right by 15 degrees in supine position. Intraoperative ultrasonography guided puncture of the right anterior hepatic duct, through which a zebra guidewire was advanced into the intrahepatic biliary system. With the guidance of the zebra guidewire, the tract was immediately expanded by the biliary expanders step-by-step until it could hold an 18-Fr protective sheath. In this way, a working channel for the rigid choledochoscopy was established.

Step 2: Choledochoscopy revealed distal obstruction of the right anterior hepatic duct, preventing guidewire passage. Then, a puncture needle was used to puncture the right anterior hepatic duct to the distal common bile duct, and a balloon was used to dilate the channel. A self-expanding metal stent was deployed, creating the first bridge. Intraoperative cholangiography confirmed successful bridging between the right anterior hepatic duct and the distal common bile duct (the first bridge).

Step 3: Under combined PTCS and ultrasound guidance, the right anterior hepatic duct was punctured into the left hepatic duct. The tract was re-dilated, and an 8 x 60 mm metal biliary stent was deployed, forming the second bridge. X-ray examination by injecting contrast agent showed successful bridging of the right anterior and left hepatic ducts (the second bridge), but poor imaging of the right posterior hepatic duct suggested that the right posterior hepatic duct may still be narrow.

**Figure 1:**
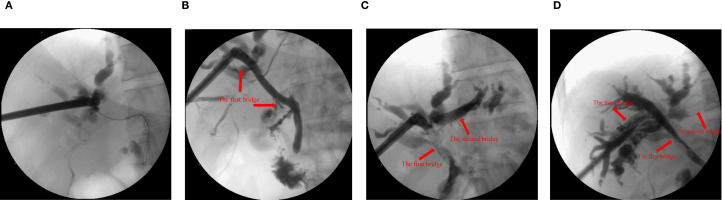
X-ray examination with contrast agent injected through the sinus. **(A)** the common bile duct before guide wire puncture and balloon dilation. **(B)** the right anterior intrahepatic bile duct and the common bile duct were bridged with metal stent (10x60 mm, the first bridge). **(C)** the left and right anterior intrahepatic bile ducts were bridged with metal stent (8x60 mm, the second bridge). **(D)** the right anterior and right posterior intrahepatic bile ducts were bridged with metal stent (8x60 mm, the third bridge).

Step 4: Finally, ultrasound-guided puncture from right anterior hepatic duct into right posterior hepatic duct. The passage between the right anterior and right posterior hepatic ducts was re-dilated using the guidewire. The right anterior hepatic duct was bridged with an 8 x 60 mm metal biliary stent to the right posterior hepatic duct. Cholangiography confirmed successful communication between the right anterior and right posterior hepatic ducts (the third bridge) (**Figure 1**).

### 2.3 Outcome

**Figure 2:**
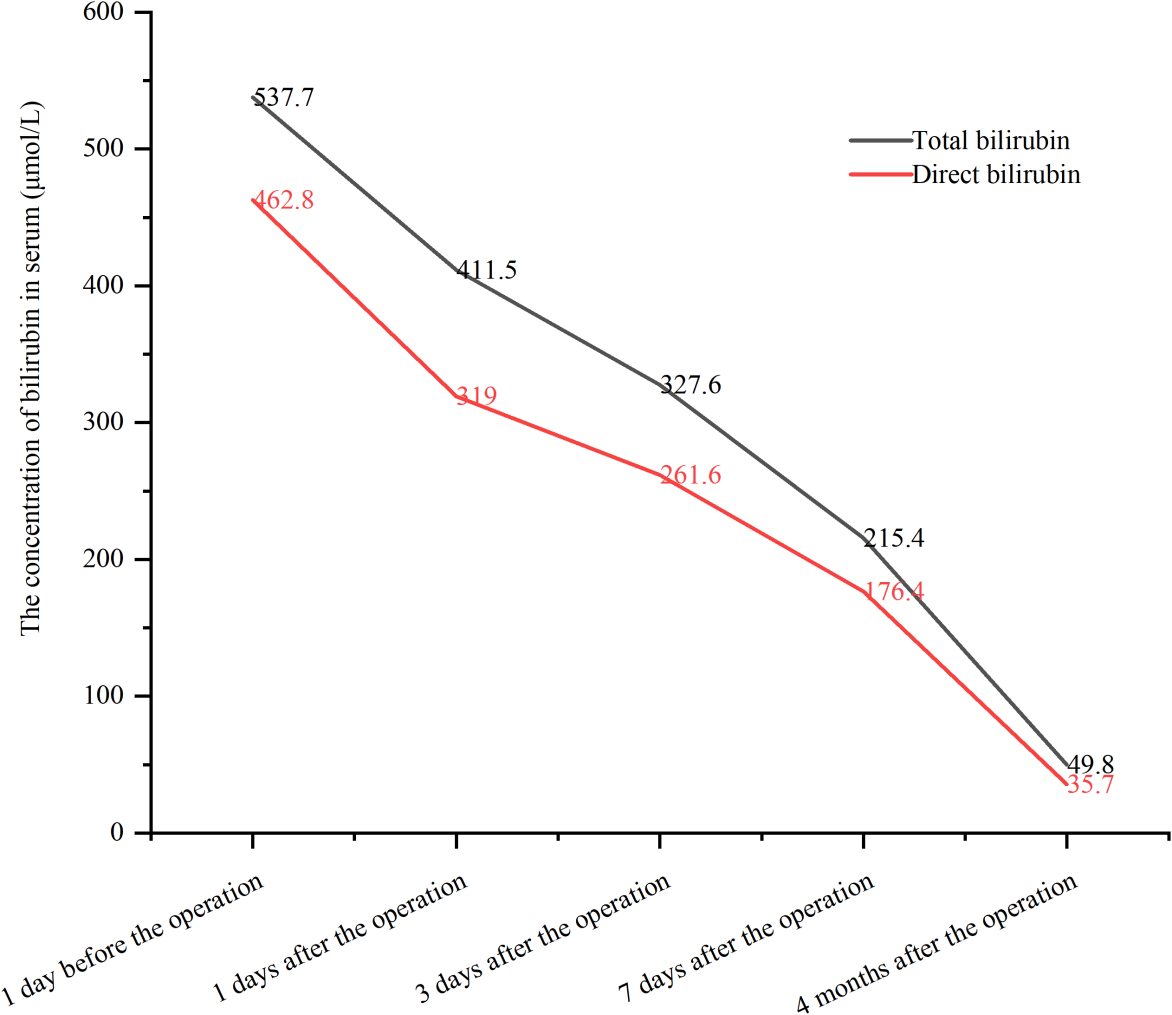
The changes of bilirubin before and after the procedure.

**Figure 3:**
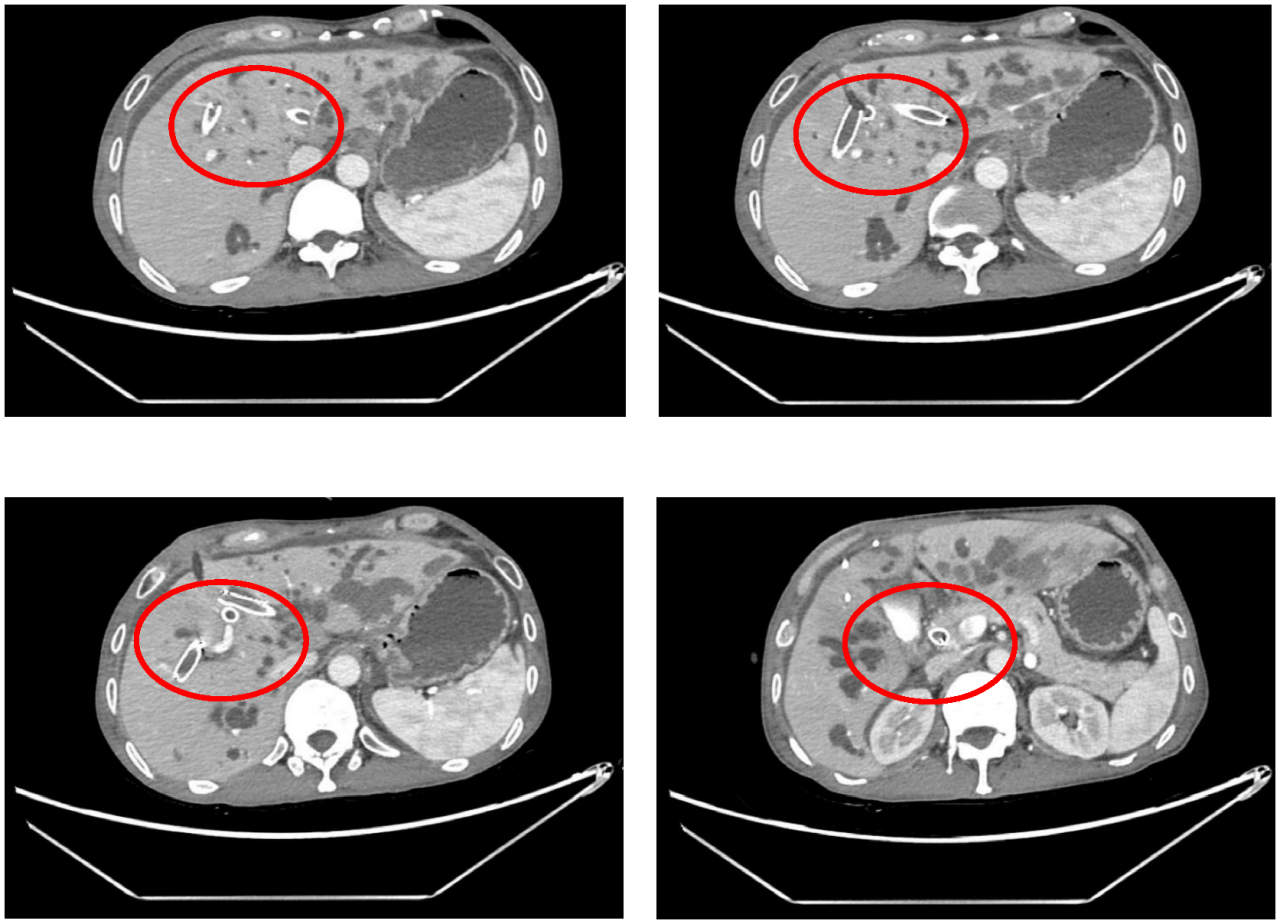
Computed tomography showed no migration of the stents.

The procedure time was 239 minutes. Jaundice resolved within several days, with total and direct bilirubin levels decreasing to 215.4 μmol/L and 176.4 μmol/L, respectively, within one week (**Figure 2**). No post-procedure-related adverse events occurred. The patient’s bilirubin decreased by 60% within one week after the procedure compared with the preoperative value, indicating clinical success. Post-procedure CT (**Figure 3**) at one week showed that bilateral hepatic duct drainage was smooth. The hospital stay was 5 days (ICU stay: 0 days). Palliative adjuvant therapy commenced three weeks post-procedure. During the four-month follow-up period, no reintervention (like another PTBD or surgery) was required.

## 3 Discussion

PTBD remains the primary palliative drainage option for MHBO when ERCP fails (11, 12). However, PTBD is limited by the small caliber of drainage catheters, which restricts drainage efficiency, and is associated with high re-intervention rates and complication risks (13–15). Páez-Carpio et al. reported a major complication rate of up to 21% (16). PTBD cannot achieve bilateral bile duct bridging, and complex MHBO often necessitates multiple drainage catheters. Additional catheters not only increase postoperative complication risk but also worsen patient discomfort. Moreover, due to biliary atresia, internal biliary drainage is often unachievable with PTBD. Compared with internal biliary drainage, external biliary drainage not only affects the nutritional status and immune function of patients, but also may damage the long-term prognosis of patients (17, 18). Kumar et al. demonstrated that internal drainage significantly improves overall survival compared with external drainage (18). Endoscopic ultrasound-guided biliary drainage (EUS-BD) has received extensive attention in recent years as a novel minimally invasive treatment method (19–22). Initially developed for distal bile duct strictures (23), technological advances have extended its application to MHBO (20, 24, 25). Compared with PTBD, EUS-BD can achieve intraluminal drainage and keep the stent away from the tumor, providing a longer stent patency time and a lower re-intervention rate (26). EUS-BD includes EUS-guided hepaticogastrostomy (EUS-HGS), EUS-guided choledochoduodenostomy (EUS-CDS) and EUS-guided hepaticoduodenostomy (EUS-HDS). EUS-guided rendezvous can be considered after ERCP failure (25). Kongkam et al. (7) reported a new strategy combining ERCP and EUS-BD for the treatment of MHBO (CERES). If the self-expandable metal stent is placed in the right biliary system, EUS-HGS can be performed subsequently. However, if the stent is placed in the left biliary system, EUS-HDS is performed. For MHBO with no function in the right liver, EUS-HGS can be performed after ERCP failure, or primary HGS can be performed in the left liver. A multicenter retrospective study (7) involving 36 patients with native high grade-MHBO demonstrated that compared with bilateral PTBD, combined ERCP and EUS-BD significantly reduced the recurrent biliary obstruction rate at 3 months and 6 months. And there was no significant difference between the two groups in terms of the incidence of complications and mortality. Its ability to achieve internal drainage offers clear advantages over PTBD, and its higher technical success rate makes it favorable compared with bilateral self-expandable metal stent via ERCP (7, 25).

Interbiliary bridging drainage has recently gained attention as a potential internal drainage strategy for complex MHBO. However, bilateral intrahepatic bridging remains rarely reported, with only four studies published to date (27–30) (**Table 1**). In 2014, Ogura et al. (27) reported the first case of EUS-guided hepaticogastrostomy (EUS-HGS) combined biliary bridge procedure. They placed two metal stents in sequence (one connecting the left and right ducts, and another for hepaticogastrostomy) to treat a patient with hilar obstruction caused by colorectal cancer metastasis. In the same year, Reimao et al. (28) reported nine patients undergoing EUS-HGS biliary bridging for MHBO. All patients were treated with three-step drainage. Step 1: EUS-guided left duct puncture with a 19-gauge needle. Step 2: Insert 0.0035 inch guide wire located on the right biliary tree where it crosses the bile duct bifurcation. After expansion, a self-expanding metal stent without cover was placed to connect the left and right bile ducts. Step 3: A second stent is inserted into the left bile duct, with the distal part in the previous stent and the proximal edge in the stomach. Drainage failed in 2/9 patients, and complications occurred in 33% (4 cases). Postoperative mortality was 8%, and 70% of patients proceeded to chemotherapy (7). In 2020, Atalla et al. (29) described a patient who had previously undergone distal gastrectomy and Roux-en-Y surgery for gastric cancer and developed postoperative liver metastases with hilar obstruction. They performed EUS-HGS bile duct bridging and common bile duct stent implantation for the patient. In addition, in 2023, Niiya et al. (30) reported a patient with gallbladder cancer who received multiple ERCP treatment due to MHBO, and was admitted to hospital again due to cholangitis after five stents were placed successively. CT showed dilated intrahepatic and right posterior bile duct (RPD) in this patient. ERCP failed because tumor obstruction prevented the punctured RPD from guiding RPD drainage from the duodenum. Therefore, they used EUS-HGS combined bile duct bridging method. Hence, bile duct bridging procedure is an important strategy for the treatment of complex MHBO. However, due to the difficulty of EUS-HGS bile duct bridging and the need to be performed by an experienced physician in a high-volume center, further promotion of this method is limited (6).

**Table 1:** Study characteristics of the studies using ultrasound-guided hepaticogastrostomy with bridging.

First author, year	Setting	Gender	Age	Sample size	Diagnose	Drainage strategy	Outcome
Ogura (2014) (27)	Japan	Female	57	1	Colon cancer with multiple liver metastases	Ultrasound-guided hepaticogastrostomy with left and right bile duct bridging	NA
Reimão (2014) (28)	France	NA	NA	9	Metastasis of a pancreatic adenocarcinoma n = 4, cholangiocarcinoma n = 1, gallbladder cancer n = 2 and metastasis from a pancreatic neuroendocrine tumor n = 2	Ultrasound-guided hepaticogastrostomy with left and right bile duct bridging	Successful drainage was observed in seven patients, two of them presented abdominal pain during the first 72 h. One patient developed sepsis and death 7 days after the procedure and the other one had drainage failure. Jaundice was reduced significatively in seven patients and a chemotherapy was started in 6/7 patients
Atalla (2020) (29)	Japan	Female	69	1	Gastric cancer with liver metastasis	Ultrasound-guided hepaticogastrostomy with left and right bile duct bridging	The patient improved clinically, with a dramatic decrease in serum bilirubin level from 9.2 mg/dL into 1.9 mg/dL, within 1 week. No procedure-related adverse events were encountered.
Niiya (2023) (30)	Japan	Female	58	1	Gallbladder carcinoma (with a history of multiple endoscopic treatments for hilar obstruction)	Ultrasound-guided hepaticogastrostomy with left and right bile duct bridging	Recovery was uneventful, and cholangitis subsided within a few days

NA, not available.

PTCS, with its ability to provide direct intraductal visualization, may overcome some limitations of EUS-HGS in interbiliary bridging. PTCS is well established in the management of intrahepatic bile duct stones and strictures (31–33) and in diagnosing malignant biliary obstruction (34), and has shown therapeutic potential in unresectable MHBO (35). PTCS-guided bridging offers several advantages. First, PTCS can be used to observe the lesions directly, and the direction could be adjusted by cholangioscope (36). After ultrasound-guided puncture, the contralateral intrahepatic bile duct can be observed by PTCS, which ensured safety of the puncture. In addition, when complications such as bleeding occur, PTCS can quickly find the bleeding point and carry out effective hemostasis. We previously reported two cases of successful bilateral drainage using PTCS combined with ultrasound-guided interbiliary bridging (37, 38). In the present case, however, multiple intrahepatic duct occlusions required more than a single bridge to achieve adequate drainage. Therefore, we carried out the first case of three-bridge connection between bile ducts and successfully achieved adequate biliary drainage. In addition, our novel technique achieves internal drainage by bridging the right anterior bile duct and the common bile duct to imitate the endoscopic method. In this study, the patient’s bilirubin decreased rapidly in the days after procedure. In addition, the CT examination one week post-procedure indicated that the three stents were in place, and the bilirubin level decreased significantly. All these suggest that our drainage strategy is effective. Previous studies have reported postoperative complication rates of PTCS ranging from 22% to 65%, with the most common adverse events being cholangitis, bleeding, pleural effusion, and bile leakage (33, 39, 40). In addition, long-term stent-related complications include re-occlusion and migration. Caillol et al. (41) reported a 33% (4/12) complication rate for EUS-bridging, with ≥ CD grade III events in 8.3% (1/12). Although our patient experienced no adverse events during 4-month follow-up, larger studies are required to determine the safety of PTCS-guided bridge biliary drainage.

In conclusion, ultrasound-guided PTCS triple-bridge biliary drainage between multiple bile ducts represents a feasible and effective strategy for complex MHBO, offering a novel internal drainage option. It is necessary to accumulate more cases to further explore the potential benefits of this approach.

## Data Availability

The original contributions presented in the study are included in the article/**Supplementary Material**. Further inquiries can be directed to the corresponding authors.

## Supplementary material

Supplementary Figure 1 Computed tomography showed advanced intrahepatic cholangiocarcinoma, hilar bile duct invasion, and radical surgery is not possible. The patient's common bile duct, left, right anterior, and right posterior hepatic ducts were obstructed.

Supplementary Video 1 We successfully performed biliary drainage of the bilateral hepatic duct system using a triple bridge drainage method from the ultrasound-guided percutaneous transhepatic cholangioscopy in malignant hilar biliary obstruction.
