# Supplementary figures and images for: Case Report: First case of percutaneous transhepatic cholangioscopy guided triple bridge drainage between multiple bile ducts for malignant hilar biliary obstruction
Source: Front Oncol. 2025 Oct 2;15:1620937. doi: 10.3389/fonc.2025.1620937 (PMC12527832; doi:10.3389/fonc.2025.1620937)

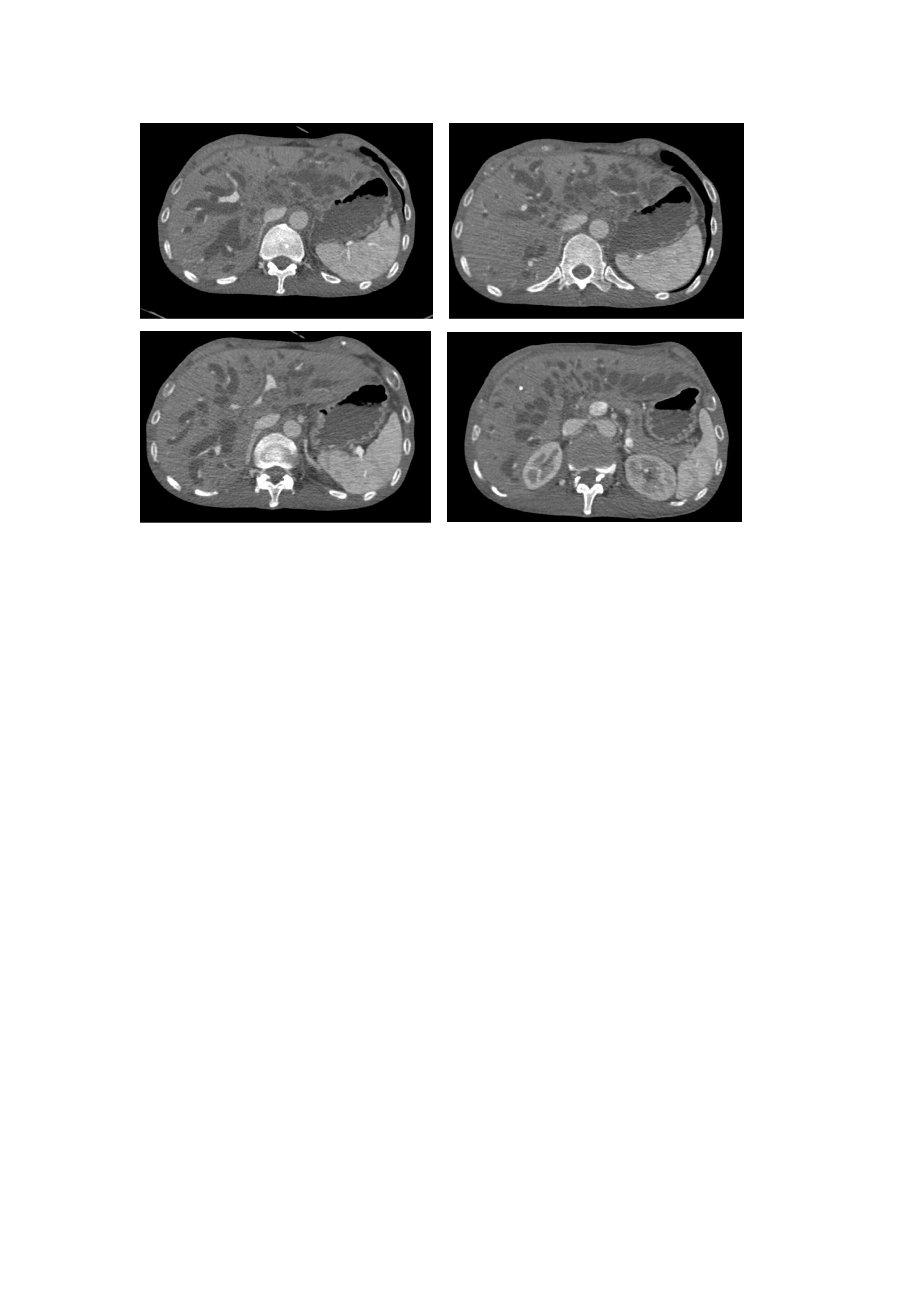

Supplement: Supplementary Figure 1 — Computed tomography showed advanced intrahepatic cholangiocarcinoma, hilar bile duct invasion, and radical surgery is not possible. The patient's common bile duct, left, right anterior, and right posterior hepatic ducts were obstructed. [file Image1.tiff]
